# Clinical challenges of glioma and pregnancy: a systematic review

**DOI:** 10.1007/s11060-018-2851-3

**Published:** 2018-04-06

**Authors:** A. van Westrhenen, J. T. Senders, E. Martin, A. C. DiRisio, M. L. D. Broekman

**Affiliations:** 1 Department of Neurosurgery, University Medical Center Utrecht, Heidelberglaan 100, 3584 CX Utrecht, The Netherlands; 2 Department of Neurosurgery, Brigham and Women’s Hospital, Computational Neuroscience Outcomes Center, Harvard Medical School, 60 Fenwood Road, Boston, MA 02115 USA; 3 Stichting Epilepsie Instellingen Nederland Heemstede, Achterweg 5, 2103 SW Heemstede, The Netherlands; 4 Department of Neurosurgery, University Medical Center Utrecht, HP G03.124, PO Box 85500, 3508 GA Utrecht, The Netherlands

**Keywords:** Glioma, Glioma patient, Pregnancy, Pregnant patient, Clinical management

## Abstract

**Introduction:**

This review aims to summarize challenges in clinical management of concomitant gliomas and pregnancy and provides suggestions for this management based on current literature.

**Methods:**

PubMed and Embase databases were systematically searched for studies on glioma and pregnancy. Observational studies and articles describing expert opinions on clinical management were included. The strength of evidence was categorized as arguments from observational studies, consensus in expert opinions, or single expert opinions. Risk of bias was assessed by the Newcastle-Ottawa Scale (NOS).

**Results:**

27 studies were selected, including 316 patients with newly diagnosed (n = 202) and known (n = 114) gliomas during pregnancy. The median sample size was 6 (range 1–65, interquartile range 1–9). Few recommendations originated from observational studies; the remaining arguments originated from consensus in expert opinions.

**Conclusion:**

Findings from observational studies of adequate quality include (1) There is no known effect of pregnancy on survival in low-grade glioma patients; (2) Pregnancy can provoke clinical deterioration and tumor growth on MRI; (3) In stable women at term, there is no benefit of cesarean section over vaginal delivery, with respect to adverse events in mother or child. Unanswered questions include when pregnancy should be discouraged, what best monitoring schedule is for both mother and fetus, and if and how chemo- and radiation therapy can be safely administered during pregnancy. A multicenter individual patient level meta-analysis collecting granular information on clinical management and related outcomes is needed to provide scientific evidence for clinical decision-making in pregnant glioma patients.

**Electronic supplementary material:**

The online version of this article (10.1007/s11060-018-2851-3) contains supplementary material, which is available to authorized users.

## Introduction

Improved prognosis in glioma patients has led to a greater number of young, female glioma patients becoming pregnant despite their diagnosis [1, 2]. No increased risk of glioma development during pregnancy has been reported [3]. However, changes in glioma behavior during pregnancy have been described, including increased tumor volume and growth rates, earlier onset of symptoms, and increased frequency of seizures [2, 4–6]. In low-grade glioma (LGG) patients specifically, dedifferentiation and tumor progression during pregnancy has been found [2, 7, 8].

Although exact incidence rates of gliomas during pregnancy are lacking, the incidence of primary malignant brain tumors in pregnant women is 2.6–15 per 100,000 [9, 10]. Due to this low incidence, evidence on clinical outcome and management of pregnant glioma patients has been based on small case series and expert opinions only. These studies include heterogeneous groups of patients and scarcely report on quality of life.

Pregnancy in glioma patients raises many difficult questions for their treatment team, ranging from: ‘What anticonvulsant therapy is least teratogenic?’ to ‘When can delivery be induced?’. Although previous studies have provided potential management algorithms for gliomas in pregnancy, the evidence is sparse, and many questions remain unanswered [2, 11]. This review aims to summarize challenges in clinical management of concomitant gliomas and pregnancy and to provide suggestions for this management based on current literature.

## Methods

This review is conducted in accordance with the Preferred Reporting Items for Systematic Reviews and Meta-Analyses (PRISMA) statement [12].

### Search and study selection

PubMed and Embase databases were systematically searched for studies on glioma and pregnancy, with the most recent search on December 3, 2016 [Supplementary Table 1]. Studies were included that evaluated clinical management in glioma patients diagnosed before or during pregnancy. Clinical management was defined as any step in the neuro-oncological treatment of glioma patients before, during or after pregnancy. Studies reporting on other brain tumors in addition to gliomas were also included, provided that they reported outcome data of glioma patients separately. Pre-clinical studies and studies not written in English were excluded. All studies that met inclusion criteria and were published in peer-reviewed journals were included, but published conference abstracts were excluded because they represent only a synopsis of scientific work that is presented in its entirety elsewhere. There were no restrictions regarding study design, in order to be as comprehensive as possible; therefore reviews and letters to the editor were also included. Eligible studies were selected by two independent authors (JS, EM) and disagreements were solved by discussion.

### Data extraction and analysis

The following data was extracted: study design, patient population, tumor characteristics, and clinical course and management during pregnancy. Topics of interest, regarding clinical management during pregnancy, were selected beforehand and correlating recommendations were extracted and assessed according to the Oxford Centre for Evidence-based Medicine (CEBM) Levels of Evidence [Supplementary Table 2] [13]. A qualitative synthesis of results was provided, and findings were summarized in a narrative fashion. Because no central histopathological review has been made, it is not feasible to perform a cross comparison by histopathological subtype and tumor grade between the studies. Risk of bias among included studies was assessed using the Newcastle-Ottawa Scale (NOS) [14].

## Results

**Fig. 1:**
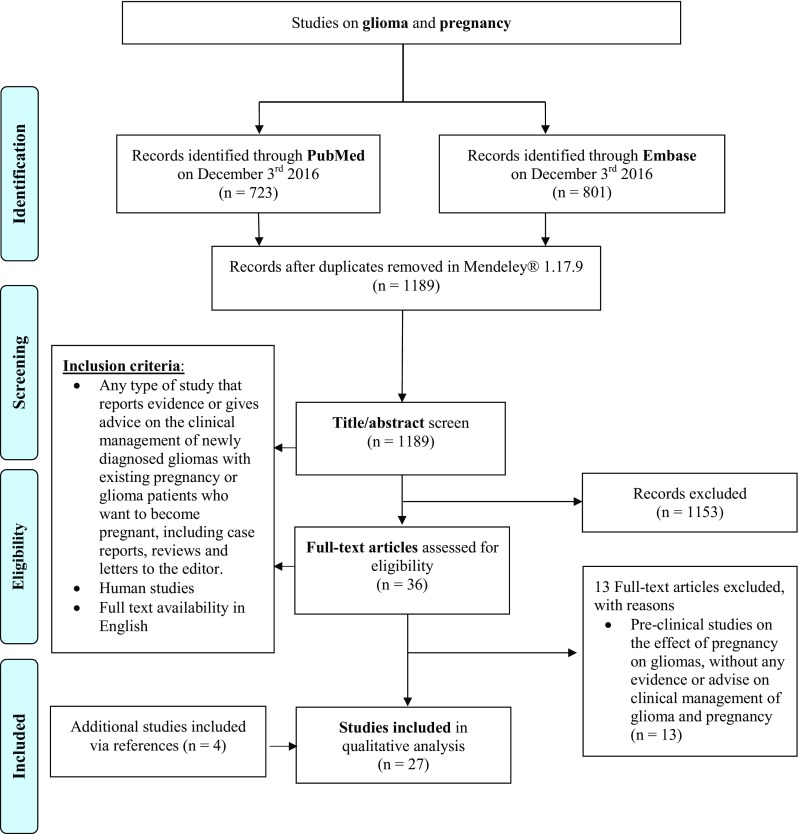
Flowchart of study search and selection

The search strategy resulted in 1189 hits after removal of duplicates, and 27 studies were eligible for data extraction (Fig. 1). Included studies were predominantly case reports (n = 9) or case series (n = 14), with a median sample size of six patients (range 1–65, interquartile range 1–9). There were three expert opinion pieces and one cohort study. In total, 316 glioma patients were included, of whom 202 were diagnosed during pregnancy, and 114 before pregnancy. Eight studies evaluated brain tumor patients in general and included 26 non-glioma brain tumor patients in addition to glioma patients (Table 1). The WHO tumor grade ranged between I and IV. Here we describe different opinions on clinical decision-making in known and newly diagnosed gliomas during pregnancy (Table 2).

**Table 1:** Characteristics of included studies

Study	Study type	Population size	Median age* [range]	Tumor type	WHO-grade	Known(K)/newly diagnosed (N)
Peeters et al. [2]	Multicenter case series	52	26.0 [19–37]	Glioma WHO II (32), glioma WHO III (14), glioma WHO IV (6)	II–IV	K (24), N (28)
Kasai et al. [34]	Case report	1	39	Oligodendroglioma	II	N **
Pallud [16]	Letter to the editor	NA	NA	NA	NA	NA
Rønning et al. [17]	Cohort study	65	31.4/25.3^††^ [16–40]	Astrocytoma (37), pilocytic astrocytoma (12), oligodendroglioma (11), oligoastrocytoma (5)	I–II	K (25), N (40)
Umehara et al. [28]	Case report	1	30	Pylocytic astrocytoma	I	K
Al-Rasheedy et al. [32]	Case report	1	36	GBM	IV	K
Taylan et al. [10]	Case report	1	21	GBM	IV	N
Abd-Elsayed et al. [25]	Case series	7	36 [29–40]	LGG (2), HGG (2), glioma (not specified) (1), GBM (1), non-glial (1)	I–IV	N
Daras et al. [8]	Case series	3	27 [15–39]	Diffuse astrocytoma WHO II (1), well differentiated astrocytoma WHO II (1), non-glial (1)	II	K
Flechl et al. [18]	Case report	1	37	GBM	IV	K
Gülşen et al. [30]	Case report	1	30	Gliosarcoma	IV	N
Yust-Katz et al. [20]	Case series	15	30/28^††^ [24–38]	Glioma WHO II (6), glioma WHO III (6), GBM (3)	II–IV	N
Wu et al. [23]	Case series	2	[25–42]	Glioma WHO III	III	N
Zwinkels et al. [6]	Case series + Retrospective cohort	7	29 [15–37]	Astrocytoma (3), oligoastrocytoma (2), ependymoma (1), pleiomorphic xanthoastrocytoma (1)	I–II	K (6), N (1)
		103	NA	LGG (53), HGG (50)	I–III	K (28), N (75)
Scarrott et al. [24]	Case report	1	32	GBM	IV	K
Lynch et al. [7]	Retrospective case series	10	33 [25–37]	Astrocytoma WHO II (3), oligodendroglioma WHO II (1), non-glial (6)	II	N
Lew et al. [22]	Letter to the editor	1	26	GBM	IV	N
Pallud et al. [5]	Case series	11	25.5 [21.5–38.5]	WHO II gliomas	II	K
Johnson et al. [15]	Case series	22	Mean 31.7/32.1^††^	Astrocytoma (5), oligodendroglioma (2), ependymomas (2), mixed glioma (2), non-glial (10)	II	K (13), N (9)
Pallud et al. [4]	Retrospective case series	8	27 [18–33]	Oligodendroglioma WHO II (4), oligodendroglioma WHO III (1), astrocytoma WHO II (1), astrocytoma WHO III (1), unknown (1)	II–III	K (5), N (3)
Blumenthal et al. [19]	Case series	6	28 [24–33]	Anaplastic astrocytoma (2), anaplastic oligodendroglioma (1), oligodendroglioma (1), oligoastrocytoma (1), GBM (1)	II–IV	N
Mackenzie et al. [27]	Case report	1	48	GBM	IV	N
Stevenson and Thompson [29]	Literature review	NA	NA	NA	NA	NA
Haba et al. [31]	Case report	1	33	Anaplastic astrocytoma	III	N
Tewari et al. [11]	Case series	8	24 [19–37]	GBM (4), anaplastic astrocytoma (3), non-glial (1)	III–IV	N
Isla et al. [26]	Case series	7	32 [28–38]	Ependymoma (2), low grade astrocytoma (2), non-glial (2), unknown (1)	II	N
Nishio et al. [21]	Retrospective case series	6	29 [23–35]	Malignant ependymoma (1), anaplastic astrocytoma (1), pilocytic astrocytoma (1), non-glial (3)	I–III	N

*LGG* low-grade glioma, *HGG* high-grade glioma, *GBM* glioblastoma multiforme, *WHO* World Health Organization, *NA* not applicable

*In years, at glioma diagnosis

**Pregnancy discovered 1 week after diagnosis. † Parity after diagnosis: 0/≥1

^††^Newly diagnosed / known glioma

**Table 2:** Considerations for clinical decision-making in pregnant glioma patients

Timing in clinical decision-making	Considerations	CEBM level of evidence
Pre-pregnancy counseling	Evidence based on observational studies	
	- Pregnancy may increase the risk of tumor growth, dedifferentiation and recurrence in glioma patients [4, 6, 8, 15]	4
	- Pregnancy does not influence overall survival in LGG [17]	4
	- Pregnancy followed by birth of a healthy infant can occur three weeks after the last chemotherapy in a glioblastoma [18]	4
	Expert opinions	
	- Use adequate pre-pregnancy counseling for all glioma patients who express the wish to become pregnant [2, 4, 6, 8, 15–17], and discuss	5
	- The possibility of tumor growth, dedifferentiation and recurrence [4, 6, 7, 15]	5
	- The benefit-to-risk ratio for mother and fetus [4]	5
	- The pros and cons of anticonvulsant therapies and neuroimaging during pregnancy [2, 15]	5
	- Counsel all female patients with LGG in their reproductive years, regardless of the decision to become pregnant [17]	5
	- Postpone pregnancy until chemo- or radiation therapy has finished [6]	5
	- Pregnancy is not recommended in patients with HGG, untreated glioma, glioma under treatment, progressive residual tumors, clinical deterioration, uncontrolled seizures or gliomas with an unfavorable molecular profile [2]	5
	- Hormonal stimulation for IVF is not recommended [6]	5
Monitoring	Expert opinions	
	- Closely follow-up of glioma patients during pregnancy, with repeated MRIs and rigorous obstetrical monitoring in a tertiary care center with multidisciplinary management for optimal neurological and obstetrical care is recommended [2, 4, 6, 15, 19, 21–23]	5
	- Apply routine follow-up MRI at 32–36 weeks of gestation, so that labor as well as tumor therapy can be scheduled [6]	5
	- Gadolinium should not be administered during pregnancy unless there is an essential clinical indication [20]	5
	- Contrast agents increase diagnostic yield and should therefore not be withheld [15]	5
Neuro-oncological treatment	Evidence based on observational studies	
	- It is safe to postpone surgery when the patient is in stable condition [22, 23, 25–27]	4
	- Surgery can be safely performed after the first trimester [10, 11, 23]	4
	- Alkylator-based chemotherapy (PCV or TMZ) administered for the treatment of a glioblastoma during the first trimester can result in the birth of a healthy infant [18]	4
	Expert opinions	
	- Indication for neurosurgery is highly dependable on tumor site, size and type, neurological signs and symptoms, gestational period and the patient’s wishes [6, 25]	5
	- Postpone surgery after the first trimester, preferably after delivery, when the patient is in stable condition [8, 22, 23, 25–27]	5
	- Surgical intervention is inevitable when condition of the patient declines with changes in mental state [2, 6, 8, 10, 25, 28, 29]	5
	- Risk of biopsy outweighs risk of inadequate treatment [8, 11, 29–31]	5
	- The second trimester seems to be the ideal timing for surgery [10, 11, 23]	5
	- Avoid nitrous oxide and isoflurance [25]—Do not perform surgery before 24 or 30 weeks of gestation [8, 25]	5
	- Apply radiation therapy only after the first trimester and minimize the scattering dose < 10 mV, with axial plane trough the head and shielding of the pelvis [11, 19, 31]	5
	- Avoid chemotherapy during the first trimester, especially PCV (probarbazine, lomustine and vincristine), carmustine (Gliadel) is presumed to be relatively safe in pregnant glioma patients [19]	5
	- Apply dorsal decubitus position, with trunk rotation to the left and avoid ventral decubitus position during surgery [8]	5
Medical treatment	Evidence based on observational studies	
	- Serum levels of anticonvulsants can fluctuate during pregnancy and should therefore be monitored closely [6, 26, 34]	4
	Expert opinions	
	- The benefit of preventing and treating epileptic seizures outweighs the teratogenic effects of anticonvulsants [8, 11, 22, 26, 27, 34]	5
	- Lamotrigine, levetiracetam and carbamazepine monotherapy should be considered as first choice [6, 8, 11]	5
	- Anticonvulsant treatment should be optimized before conception [19]	5
	- After delivery, the anticonvulsant dosages should be re-adjusted to the original dose within two to three days [6]	5
	- When prescribing anticonvulsants that interfere with vitamin K absorption, treat mother and born child with vitamin K [19]	5
	- Apply corticosteroids during the second and third trimester of pregnancy [8, 11, 25, 27]	5
	- Avoid chronic use of dexamethasone during pregnancy [25, 26]	5
	- Prescribe prednisolone when lung maturation is not preferred [31]	5
	- Betamethasone is preferred over dexamethasone, because of fewer side effects [10]	5
Obstetrical treatment	Evidence based on observational studies	
	- No benefit of CS over vaginal delivery is described, when the mother is at term and in stable condition [2, 5, 11, 25, 27]	4
	Expert opinions	
	- CS should be performed at fetal maturation, when the patient’s condition declines and, if the patient is stable enough, before tumor resection [22, 23, 28]	5
	- Delivery at 34–36 weeks of gestation is preferable [21, 23]	5
	- Apply CS under general anesthesia to reduce risk of cerebral herniation [6, 11, 16, 27]	5
	- Apply CS in nulliparous women, because of their risk of increased intracranial pressure during delivery [21]	5
	- Apply CS and tumor resection under the same general anesthesia to minimize the risk for mother and child [11, 23]	5
	- Apply CS and tumor resection in two different sessions to allow for maternal-fetal bonding [27]	5
	- Avoid mannitol and hypocapnia to prevent fetal dehydration and cerebral ischemia respectively [25]	5
	- Epidural anesthesia can be given to shorten the second phase of delivery and decrease risk of increased intracranial pressure [27]	5
	- After delivery, treat the women as a non-pregnant patient, according to the WHO-treatment criteria [2, 21]	5

CEBM (Oxford Centre for Evidence-based Medicine—Levels of Evidence), 4: Case series and poor quality cohort studies, 5: Expert opinion

*LGG* low-grade glioma, *HGG* high-grade glioma, *CS* Cesarean section, *PCV* probarbazine, lomustine and vincristine, *TMZ* temozolomide

### Pre-pregnancy counseling

Seven studies emphasize the importance of adequate counseling for glioma patients who express interest in becoming pregnant [2, 4, 6, 8, 15–17]. The exact content remains ambiguous because it is challenging to predict the outcomes associated with pregnancy in an individual case with sufficient accuracy.

One of the primary concerns is that pregnancy will trigger tumor growth and negatively influence prognosis of glioma patients, but there is no evidence to support this [2]. The largest cohort study included in this review did not find any evidence to suggest that overall survival in LGG is affected by pregnancy [17]. However, changes in tumor behavior, including tumor growth and dedifferentiation, have been described [4, 5]. A case series of eight patients with diffuse glioma observed unfavorable tumor evolution in six cases during pregnancy [4]. Three out of five gliomas diagnosed before pregnancy presented with clinical worsening, increase in tumor growth, and change in histopathological grade [4]. A multicenter case series with different WHO-grade gliomas found clinical deterioration in 37.5% of cases during pregnancy and significant tumor progression on MRI, compared with pre-pregnancy values [2]. High-grade gliomas and gliomas with unfavorable molecular profile were particularly likely to be associated with tumor progression during pregnancy [2]. Some authors suggest discussing the risks and benefits of neuroimaging [15] and anticonvulsant therapies [2] before pregnancy.

### Timing of pregnancy

Although teratogenicity of chemotherapy has not yet clearly been demonstrated, it is advisable to postpone pregnancy until after administration of these therapies [6]. The best timing of conception remains unclear, but a case report has shown that pregnancy and the birth of a healthy infant can occur as early as 3 weeks after the last chemotherapy and even after heavy pretreatment with alkylating agents [18].

Zwinkels et al. mentioned assisted reproductive technology in female glioma patients, but discouraged it since hormonal stimulation, using progesterone and gonadotropin hormones, could induce tumor growth and dedifferentiation [6].

### Monitoring

Although no statement is made on the exact frequency of neurological and imaging monitoring, several studies agree that gliomas should be followed-up closely during pregnancy with repeated MRIs [2, 4–6, 15, 19]. Interestingly, no distinction in recommendations is made between different glioma WHO grades, despite their variability in treatment regimens and prognosis. Zwinkels et al. emphasize the advantages of a routine follow-up MRI at 32–36 weeks of gestation, since both induction of labor, as well as tumor therapy, can be initiated at this point if indicated [6]. No deleterious consequences of MRI or CT neuroimaging have been reported, but iodine compounds of CT contrast agents might induce fetal hypothyroidism [15]. Some argue that gadolinium should not be administered during pregnancy unless there is an essential clinical indication [20], while others state that contrast agents increase diagnostic yield and should therefore not be withheld [15]. Rigorous obstetrical monitoring is advised, but no detailed guidelines are provided [5, 6, 21]. Five studies indicate the importance of multidisciplinary management at a tertiary care center for optimal neurological and obstetrical care [10, 15, 22–24]. Prenatal screening has not been addressed in any study.

### Neuro-oncological treatment options and timing

#### Surgery

Indication for neurosurgery highly depends on tumor location, size, type, neurological signs and symptoms, gestational period, and the patient’s preferences [6, 25]. If the patient is in a stable condition, several authors suggest to postpone surgery at least until after the first trimester [22, 23, 25–27], or even after 24 [25] or 30 [7] weeks of gestation, to permit gestational advancement. If there is a clear indication for surgery, the second trimester seems to be the ideal time, since the fetus may be too vulnerable during the first, and the risk of intraoperative hemorrhage is increased during the third trimester [10, 11, 23]. Surgical intervention seems inevitable when the neurological condition of the patient declines and signs of intracranial hypertension or changes in mental state occur [2, 6–8, 25, 28, 29]. In newly diagnosed brain tumors during pregnancy, prompt tissue diagnosis via tumor biopsy is recommended by multiple groups. The risk of biopsy outweighs the risk of inadequate treatment [7, 11, 29–31]. To prevent aorto-caval compression during surgery, a dorsal decubitus position, with a trunk rotation to the left is advised, and ventral decubitus position should be avoided [7].

#### Anesthesia

It is controversial whether many anesthetic agents can be used safely during pregnancy. Some suggest avoiding nitrous oxide and isoflurance, because several case series showed their possible teratogenicity [25]. The neonatal neurotoxic effect of desflurance and sevoflurance needs to be further examined [25]. Propofol seems to be relatively safe as the main side effect is relaxing of the uterus, but seizures, ataxia, and hallucinations have been reported in the newborn when exposure exceeded six hours [25]. Whether it is necessary to monitor a fetus intraoperatively when viability is reached, remains a matter of debate [15].

#### Radiotherapy

In glioma patients, the fetus can be exposed to radiation by scattering [21]. Especially during the first trimester, therapeutic cerebral irradiation can cause serious harm to the fetus and should, therefore, be avoided [15, 22, 25, 26]. Radiation doses > 50 cGy pose a risk to the fetus during all trimesters [19]. Authors advise to calculate and minimize the scattered dose by using coplanar beams < 10 mV, with an axial plane through the head, and additional shielding of the pelvis [11, 19, 31].

#### Chemotherapy

Evidence from animal and epidemiological studies suggest that chemotherapy can cause serious teratogenicity, especially during the first trimester [7, 29, 32]. A review on chemotherapy exposure during pregnancy found chemotherapy-associated malformations in 9 out of 71 (12.7%) children, which is more than four times higher compared to the general population [33]. High risks of fetal malformation after PCV (procarbazine, lomustine, and vincristine) chemotherapy exposure during the first trimester are described [19]. The use of carmustine wafers (Gliadel) are presumed to be relatively safe in pregnant glioma patients, but no evidence was reported on systemic use of carmustine [19]. Evidence is poor and information on long-term effects on the child is lacking [6]. A case series of six malignant glioma patients with chemotherapy exposure during the first trimester, however, showed delivery of healthy babies [19]. As long as there is no consensus on the actual risk of chemotherapy during pregnancy, authors recommend to avoid it if there is no strong indication [7, 29].

### Medical treatment options (other than chemotherapeutic agents) for symptom reduction/prevention

#### Anticonvulsants

Convulsive seizures in pregnant glioma patients can cause hypoxia and fetal acidosis. Several authors agree that the benefit of preventing and treating these seizures properly, outweighs the teratogenic effects of anticonvulsants [7, 11, 22, 26, 27, 34]. Lamotrigine, levetiracetam, and carbamazepine monotherapy are suggested to be favorable, because of their low teratogenic risk. Long term side effects of levetiracetam and lamotrigine, however, are insufficiently examined [6, 7, 11]. Several groups advise monitoring serum levels of anticonvulsants, since they can fluctuate during pregnancy [6, 26, 34]. The current American Academy of Neurology guidelines states that the anticonvulsant treatment should be optimized before conception, and vitamin K supplements should be given to mother and child if the choice of anticonvulsants is one which impairs vitamin K absorption [19]. After delivery, it is advised to re-adjust the anticonvulsant dosages to the original dose within 2–3 days [6].

#### Corticosteroids

Several authors encourage the administration of corticosteroids during the second and third trimester to promote fetal lung maturation [7, 11, 25, 27]. Prednisolone is advised when lung maturation is not a priority, [31] and dexamethasone is considered safe in the acute setting [25, 26]. For chronic use, betamethasone is preferred over dexamethasone, because of a better side effect profile [10].

#### Anticoagulants

Especially in high-grade glioma patients, the risk of venous thromboembolism (VTE) is increased [35]. Pregnancy itself also increases risk of VTE, so pregnant glioma patients may benefit from prophylactic anticoagulation, but specific guidelines are lacking [36, 37].

### Obstetrical treatment options and timing

To decrease the risk of increased intracranial pressure (ICP) during delivery, some recommend the use of epidural anesthesia [27], whereas others advise a cesarean section (CS) over vaginal delivery in nulliparous women [21]. This beneficial effect of CS has not been proven in literature [2, 5, 12, 25]. Two case series did not find altered child development or adverse clinical outcome of the mother, related to the method of delivery [2, 5]. Therefore, several authors agree that there is no clinical benefit of CS over vaginal delivery if the mother is at term and in stable condition [2, 5, 11, 25, 27].

The timing of delivery depends on individual risks of the effect of pregnancy on the tumor and benefits of fetal maturation. If the patient is stable, authors advise to induce delivery at fetal maturation, and if possible, before neurosurgery [22, 23, 28]. A gestational age of 34–36 weeks has been proposed as ideal timing [21, 23]. CS at an earlier stage could, however, provide more time for adjuvant treatment [27]. If the patient deteriorates with signs of increased ICP and the child is viable, most agree to perform a CS [15, 19, 20, 25, 27]. CS under general anesthesia is recommended because this reduces the risk of cerebral herniation [6, 11, 15, 27]. Some studies advise performing CS and tumor resection under the same general anesthesia to minimize the risk of cerebral herniation and anesthesia for mother and child, [11, 23] while another study recommends performing two different sessions to allow for maternal-fetal bonding following the delivery [27].

After delivery, it is advised to treat the woman as a non-pregnant patient, according to the WHO-treatment criteria, and to discuss breastfeeding, contraceptives, and adaptation of anticonvulsants [2, 21].

### Risk of bias among included studies

According to the NOS score, four studies were at low risk of bias [Supplementary Table 3] [2, 17, 19, 21]. All other included studies were at high risk of bias, mainly due to low generalizability of the exposed cases and lack of a control group.

## Discussion

Gliomas during pregnancy are rare, but it is important to have an adequate treatment strategy. The majority of recommendations included in this review originate from expert opinions without supporting evidence from observational studies (Table 2). Rønning et al. (2016) performed a cohort study with low risk of bias and observed no association between pregnancy and survival in LGG patients. Peeters et al. [2] performed a multicenter case series with low risk of bias and found (1) Clinical deterioration and tumor progression on MRI of gliomas during pregnancy and (2) No observed benefit of CS over vaginal delivery with respect to adverse events, in stable women at term. These observations were also found in smaller case series with a high risk of bias [4–6, 11, 15, 25, 27]. All other recommendations and conclusions are reported in studies with a high risk of bias and should be interpreted with caution.

Other cancer types raise similar questions for the treatment team during pregnancy, and especially long-term outcomes of children with prenatal exposure to cancer and treatment are lacking. A multicenter, prospective case-control study involving 129 children with prenatal exposure to different types of cancer and treatment, reported no significant difference in neurocognitive and cardiac outcome, compared to matched controls [38]. The estimated fetal radiation dose and number of chemotherapy cycles were not related to the children’s outcome. However, median follow-up was 22 months, which does not reveal long-term developmental problems, chemotherapy was only administered after the first trimester, and no new targeted therapies were applied [38]. Another cohort study found a higher risk of preterm labor and small-for-gestational-age children after fetal chemotherapy exposure [39]. Both studies reported no increased incidence of congenital malformations after chemotherapy during pregnancy [38, 39]. The Physician Data Query (PDQ) guideline for breast cancer treatment and pregnancy advises to avoid chemotherapy and radiotherapy in the first trimester and to minimize scattering radiation dose to the fetus, which is in line with our findings [40]. After the first trimester, many chemotherapeutic drugs are safe to administer, but trastuzumab is contraindicated, according to the PDQ [40].

One in 200 women who attend prenatal clinics are receiving anticonvulsant treatment [41]. A large cross-sectional observational study from the United Kingdom examined the effect of monotherapy epilepsy treatment during pregnancy [42]. Lamotrigine and levetiracetam were associated with the lowest risk of congenital malformations (e.g. spina bifida and cardiac anomalies), whereas valproate was associated with the highest risk [42]. These results were confirmed by a meta-analysis on the safety of anti-epileptic drugs for neurological development in exposed children, which showed that only valproate was significantly associated with developmental delay [43]. A systematic review including eight studies on levetiracetam exposure in the second and third trimester could not find an increased overall risk of major malformation or adverse events on long-term child development, compared to the general population [44]. Two cohort studies on lamotrigine use during pregnancy reported no significant findings regarding the incidence of neurodevelopmental disorders [45, 46]. The above findings are in line with the findings from our review.

Specific evidence-based guidelines for the use of general anesthesia during pregnancy are lacking, but it is the consensus to perform surgery under general anesthesia when an untreated condition poses a high risk to mother or child. Although adverse events are rarely reported in the literature, one study showed that surgery during the first trimester might increase the risk of congenital malformations or miscarriage [47].

### Limitations

Most findings presented in this review are based on low empirical evidence. The included studies are often limited by small sample sizes, high risk of bias, and a low level of evidence. Multiple recent studies are from the same French glioma study group, which may have influenced the findings.

Many aspects of the clinical management of pregnant glioma patients remain a topic of debate [Supplementary Table 4]. Unanswered questions address issues as to when pregnancy should be discouraged, what the best monitoring schedule is for both mother and fetus, if and how chemo- and radiation therapy can be safely administered during pregnancy, and what the best delivery mode is. Moreover, it’s important to examine the long-term effects of different treatment strategies on the child’s well-being. Therefore, an individual patient-level meta-analysis is needed to investigate the conclusions and recommendations made by individual studies. Additionally, multicenter prospective registries are required that collect granular information on clinical management and related outcomes. These efforts can provide the necessary evidence for clinical decision-making in pregnant glioma patients.

## Conclusion

Clinical decision-making in glioma and pregnancy remains a great challenge for the treatment team. Very few findings are based on clinical observations originating from case series or cohort studies with low risk of bias. These include: (1) There is no known effect of pregnancy on survival in LGG patients; (2) Pregnancy can provoke clinical deterioration and tumor growth on MRI; (3) There is no benefit of CS over vaginal delivery with respect to adverse events in mother or child among stable pregnant women at term. Most recommendations originate from expert opinions or case reports and should be interpreted with caution. An individual patient data meta-analysis, as well as the inclusion of obstetric variables in (neuro-)oncological registries, could facilitate evidence-based decision-making in pregnant glioma patients.

## Electronic supplementary material

Below is the link to the electronic supplementary material.

Supplementary material 1 (DOCX 15 KB)

Supplementary material 2 (DOCX 28 KB)

Supplementary material 3 (DOCX 17 KB)

Supplementary material 4 (DOCX 15 KB)
