# Supplementary material for: Clinical challenges of glioma and pregnancy: a systematic review
Source: J Neurooncol. 2018 Apr 6;139(1):1–11. doi: 10.1007/s11060-018-2851-3 (PMC6061223; doi:10.1007/s11060-018-2851-3)
Supplement: Supplementary file 1 — Supplementary material 1 (DOCX 15 KB) [file 11060_2018_2851_MOESM1_ESM.docx]

| **Search in PubMed on December 3^rd^ 2016** |
| --- |
| ("Reproductive Health"[Mesh] OR "Pregnancy"[Mesh] OR "Gravidity"[Mesh] OR Reproductive Health[tw] OR pregnancy[tw] OR gravidity[tw] OR pregnancies[tw] OR pregnant[tw] OR gestation[tw] AND  ("Glioma"[Mesh:NoExp] OR "Glioblastoma"[Mesh] OR "Astrocytoma"[Mesh] OR "Oligodendroglioma"[Mesh] OR Glioblastoma[tw] OR GBM[tw] OR Astrocytoma*[tw] OR glioma*[tw] OR astroglioma*[tw] OR oligoastrocytoma*[tw] OR oligodendroglioma*[tw]) |
| **Search in Embase on December 3^rd^ 2016** |
| ('reproductive health'/exp OR 'pregnancy'/exp OR ‘Reproductive Health’:ab,ti OR pregnancy:ab,ti OR gravidity:ab,ti OR pregnancies:ab,ti OR pregnant:ab,ti OR gestation:ab,ti) AND  ('glioma'/de OR 'astrocytoma'/de OR 'glioblastoma'/exp OR 'oligodendroglioma'/exp OR Glioblastoma:ab,ti OR GBM:ab,ti OR Astrocytoma*:ab,ti OR glioma*:ab,ti OR astroglioma*:ab,ti OR oligoastrocytoma*:ab,ti OR oligodendroglioma*:ab,ti) |

**Supplementary table 1:** Search strategy in PubMed and Embase.
