# Supplementary material for: Clinical challenges of glioma and pregnancy: a systematic review
Source: J Neurooncol. 2018 Apr 6;139(1):1–11. doi: 10.1007/s11060-018-2851-3 (PMC6061223; doi:10.1007/s11060-018-2851-3)
Supplement: Supplementary file 3 — Supplementary material 3 (DOCX 17 KB) [file 11060_2018_2851_MOESM3_ESM.docx]

| **Study** | **Selection**  (max 4 *) | **Comparability**  (max 2 *) | **Outcome**  (max 3 *) | **Total** |
| --- | --- | --- | --- | --- |
| Peeters^2^ ‘17 | *** | - | ** | 5/6 |
| Kasai^34^ ‘16 | ** | - | * | 3/5 |
| Rønning^17^ ‘16 | **** | ** | *** | 9/9 |
| Umehara^28^ ‘16 | ** | - | * | 3/5 |
| Al-Rasheedy^32^ ‘15 | ** | - | ** | 4/5 |
| Taylan^10^ ‘15 | ** | - | * | 3/5 |
| Abd-Elsayed^25^ ‘14 | ** | - | * | 3/6 |
| Daras^8^ ‘14 | ** | - | * | 3/6 |
| Flechl^18^ ‘14 | ** | - | ** | 4/5 |
| Gülşen^30^ ‘14 | ** | - | * | 3/5 |
| Yust-Katz^20^ ‘14 | ** | - | ** | 4/6 |
| Wu^23^ ‘13 | ** | - | * | 3/6 |
| Zwinkels^6^ ‘13 | *** | - | * | 4/6 |
| Scarrott^24^ ‘12 | ** | - | ** | 4/6 |
| Lynch^7^ ‘11 | ** | - | * | 3/5 |
| Pallud^5^ ‘10 | ** | - | ** | 4/6 |
| Johnson^15^ ‘09 | * | - | * | 2/6 |
| Pallud^4^ ‘09 | ** | - | ** | 4/6 |
| Blumenthal^19^ ‘08 | ** | - | *** | 5/6 |
| Mackenzie^27^ ‘05 | ** | - | ** | 4/5 |
| Haba^31^ ‘04 | ** | - | ** | 4/5 |
| Tewari^11^ ‘00 | ** | - | * | 3/6 |
| Isla^26^ ‘97 | * | - | *** | 4/6 |
| Nishio^21^ ‘96 | ** | - | *** | 5/6 |

**Supplementary table 3: Newcastle Ottawa Quality Assessment Scale**^14^ **(NOS) score of included cohort studies, case series and case reports***Selection (4 stars): 1. Representative of the exposed cohort; 2. Selection of the non-exposed cohort; 3. Ascertainment of exposure; 4. Demonstration that outcome of interest was not present at start of study. Comparability (2 stars): 1. Comparability of cohorts on the basis of the design or analysis. Outcome (3 stars): 1. Assessment of outcome; 2. Was follow-up long enough for outcomes to occur; 3. Adequacy of follow-up of cohorts.*
*The items ‘selection of the non-exposed cohort’ and ‘comparability’ could only be assessed in cohort studies with a control group. Adequacy of follow-up could not be assessed in case reports.*
