# Supplementary material for: Clinical challenges of glioma and pregnancy: a systematic review
Source: J Neurooncol. 2018 Apr 6;139(1):1–11. doi: 10.1007/s11060-018-2851-3 (PMC6061223; doi:10.1007/s11060-018-2851-3)
Supplement: Supplementary file 4 — Supplementary material 4 (DOCX 15 KB) [file 11060_2018_2851_MOESM4_ESM.docx]

| **Insufficiently addressed topics** | **Possible strategy for further research** |
| --- | --- |
| The effect of pregnancy on the prognosis of different WHO-grade glioma patients. | Large neuro-oncological registries with outcome data on gliomas of different WHO-grade. |
| Ideal timing of neurosurgery in pregnant glioma patients without critical neurological conditions. | - Multicenter prospective observational studies on outcome of pregnant glioma patients and their children compared to timing of neurosurgery.  - Individual patient-level meta-analysis on timing of neurosurgery in pregnant glioma patients. |
| Long-term outcomes of children with prenatal exposure to chemotherapy and radiation therapy. | Multicenter prospective case-control studies or registries reporting on long-term outcomes of children after prenatal exposure. (Minimal follow-up of 10 years) |
| Risks and benefits of prophylactic anticoagulation therapy in pregnant glioma patients. | - Multicenter prospective case-control studies on outcome of pregnant glioma patients and their children after prophylactic anticoagulant treatment during pregnancy.  - Individual patient-level meta-analysis on prophylactic anticoagulant therapy in pregnant glioma patents. |
| Ideal delivery mode for pregnant glioma patients. | - Multicenter prospective observational studies on outcome of glioma patients and their children compared to delivery modes (vaginal delivery vs cesarean section).  - Individual patient-level meta-analysis on outcome of patients and their children after different modes of delivery in pregnant glioma patients. |

***Supplementary Table 4: Insufficiently addressed topics in current literature with high clinical impact and suggestions for further research.***
